# Reduction of Cardio-Metabolic Risk and Body Weight through a Multiphasic Very-Low Calorie Ketogenic Diet Program in Women with Overweight/Obesity: A Study in a Real-World Setting

**DOI:** 10.3390/nu13061804

**Published:** 2021-05-26

**Authors:** Elena Tragni, Luisella Vigna, Massimiliano Ruscica, Chiara Macchi, Manuela Casula, Alfonso Santelia, Alberico L. Catapano, Paolo Magni

**Affiliations:** 1 Dipartimento di Scienze Farmacologiche e Biomolecolari, Università degli Studi di Milano, 20133 Milan, Italy; elena.tragni@unimi.it (E.T.); massimiliano.ruscica@unimi.it (M.R.); chiara.macchi@unimi.it (C.M.); manuela.casula@unimi.it (M.C.); the.alfsan@gmail.com (A.S.); alberico.catapano@unimi.it (A.L.C.); 2 Center of Obesity and Work EASO Collaborating Centers for Obesity Management, Occupational Health Unit, Fondazione Ca’ Granda Ospedale Maggiore Policlinico, 20122 Milan, Italy; luisella.vigna@policlinico.mi.it; 3 IRCCS MultiMedica, Sesto S. Giovanni, 20099 Milan, Italy

**Keywords:** very-low calorie ketogenic diet, obesity, cardiovascular risk, insulin resistance, nutraceutical

## Abstract

Background: The prevention and treatment of obesity and its cardio-metabolic complications are relevant issues worldwide. Among lifestyle approaches, very low-calorie ketogenic diets (VLCKD) have been shown to lead to rapid initial weight loss, resulting in better long-term weight loss maintenance. As no information on VLCKD studies carried on in a real-world setting are available, we conducted this multi-centre study in a real-world setting, aiming at assessing the efficacy and the safety of a specific multiphasic VLCKD program in women with overweight or obesity. Methods: A multi-center, prospective, uncontrolled trial was conducted in 33 outpatient women (age range 27–60 y) with overweight or obesity (BMI: 30.9 ± 2.7 kg/m^2^; waist circumference: 96.0 ± 9.4 cm) who started a VLCKD dietary program (duration: 24 weeks), divided into four phases. The efficacy of VLCKD was assessed by evaluating anthropometric measures and cardiometabolic markers; liver and kidney function biomarkers were assessed as safety parameters. Results: The VLCKD program resulted in a significant decrease of body weight and BMI (−14.6%) and waist circumference (−12.4%). At the end of the protocol, 33.3% of the participants reached a normal weight and the subjects in the obesity range were reduced from 70% to 16.7%. HOMA-IR was markedly reduced from 3.17 ± 2.67 to 1.73 ± 1.23 already after phase 2 and was unchanged thereafter. Systolic blood pressure decreased after phase 1 (−3.5 mmHg) and remained unchanged until the end of the program. Total and LDL cholesterol and triglycerides were significantly reduced by VLCKD along with a significant HDL cholesterol increase. Liver, kidney and thyroid function markers did not change and remained within the reference range. Conclusions: The findings of a multi-center VLCKD program conducted in a real-world setting in a cohort of overweight/obese women indicate that it is safe and effective, as it results in a major improvement of cardiometabolic parameters, thus leading to benefits that span well beyond the mere body weight/adiposity reduction.

## 1. Introduction

Prevention and treatment of obesity and its cardio-metabolic complications are growing public health problems worldwide since this condition affects a relevant part of the world population across both genders and all ages and ethnic groups, and its prevalence is now maintained or even accelerated in most industrialized countries [1,2,3,4]. In recent years, the prevalence of obesity has reached epidemic proportions, and, therefore, the identification of effective lifestyle tools, including nutritional ones [5], able to produce significant weight loss and to maintain it over time is mandatory, in order to limit its progression from the uncomplicated stage to that characterized by cardiovascular and metabolic complications [6,7,8], as well as oncologic diseases [9]. In this context, cardiovascular disease (CVD) risk and unhealthy lifestyle habits [10] are often underdiagnosed and undertreated, therefore highly contributing to atherosclerotic CVD (ASCVD) prevalence [11]. The current treatment options for obesity include balanced hypocaloric diets, exercise, lifestyle modifications, drugs, use of endoscopic devices (e.g., intragastric balloon) and bariatric surgery [12,13,14,15]. The therapeutic benefit of all currently available anti-obesity interventions is often limited by their subjective efficacy, variable tolerability, safety profiles and poor compliance, with the latter being a strongly limiting variable, especially when long-term treatments are needed [4,16]. Many dietary regimens that operate through various mechanisms have been proposed to reduce appetite or for weight control [17,18] and the leading non-pharmacological approach is the use of diets, particularly low-calorie and very low-calorie ketogenic diets (VLCKD) [19,20,21]. VLCKD has been endorsed by the European Food Safety Agency (EFSA) for reduction of body weight in subjects with obesity, according to a specific Scientific Opinion (https://www.efsa.europa.eu/it/efsajournal/pub/2271, accessed 2 April 2021), and a specific consensus statement discussing the appropriate use of VLCKD has been recently published by a scientific panel of the Italian Society of Endocrinology [22]. Indeed, in studies conducted in hospital settings, the VLCKD approach has been shown to lead to a rapid initial weight loss, which results in better long-term weight loss maintenance [23] although in some cases an adequate weight reduction at the beginning of the diet program is followed by a shotdown of weight decrease. This problem may depend upon different factors, including individual metabolic rate and patient compliance. The VLCKD approach generally includes an initial phase with a complete replacement of regular meals with food or formulations that provide 400–800 kCal/day. This type of diet may be better defined as a “therapeutical approach” since it is commonly followed under medical supervision in patients with BMI > 30 kg/m^2^ or in subjects needing a rapid weight loss in preparation to other medical procedures [21,24] and is usually associated with the use of specific food supplements. Since, to our best knowledge, no information on VLCKD studies conducted in a real-world setting are available, the present multi-centre study, conducted in a real-world setting, was aimed at assessing the efficacy, according to anthropometric and cardiometabolic changes, and safety of a specific multiphasic VLCKD program in women with overweight or obesity.

## 2. Materials and Methods

### 2.1. Study Design and Population

**Figure 1:**
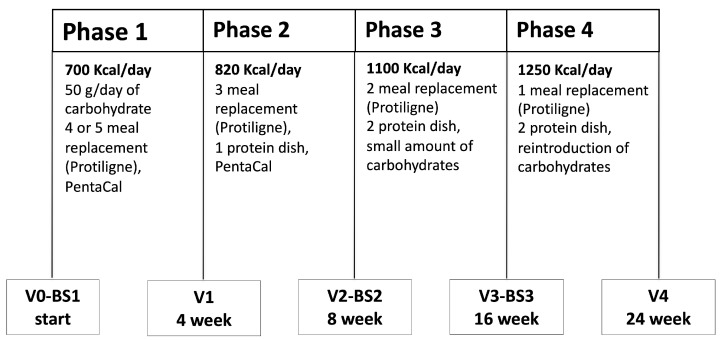
Outline of the VLCKD program. The program included 4 separate phases, with a total duration of 24 weeks.

The study was designed as a multi-center, prospective, uncontrolled trial in a real-life setting and included Caucasian outpatient women with overweight or obesity and some features of the metabolic syndrome, including increased waist circumference (WC) and pharmacologically controlled arterial hypertension [25]; 11/33 subjects were on pharmacological therapy for arterial hypertension (Table S1). All patients were consecutively admitted to one of the 5 participating clinical centers in the Milan area (Italy) in the period 2016–2018. Each clinical center is specialized in the medical management of obesity, with a specific expertise in VLCKD program, and includes expert physicians; 2 centres also included a trained dietician. The inclusion criteria were: female sex upper-range overweight or grade 1 or 2 obesity (body mass index (BMI) range: 27–37 kg/m^2^), age between 25 and 65 years, negative for pregnancy test, and having signed an informed consent. The main exclusion criteria were: current or previous smoking, pregnancy and nursing, history of diabetes mellitus, renal disease or severe renal impairment (plasma creatinine >1.5 mg/dL), severe liver disease, HIV infection, nervous system and cardiovascular diseases (including uncontrolled arterial hypertension), blood diseases, cancer or any progressive severe disease, osteoporosis, eating disorders or any psychiatric disease, uncontrolled thyroid diseases, menopause hormonal replacement therapy, pharmacological treatments known to interfere with the study treatment, history of bariatric surgery, and patients who were enrolled in another research study in the last 12 months. At the screening visit, all patients underwent fasting blood sampling and a full clinical examination, to evaluate height (in standing position and without shoes and corrected to the closer 0.5 cm), body weight, WC and hip (HC) circumferences (in standing position, measured with a flexible tape), heart rate (HR) and arterial blood pressure. These parameters were also recorded at all subsequent visits. A total of 44 eligible patients (age 49.5 ± 7.2 yrs, and BMI 30.9 ± 2.7 kg/m^2^ (mean ± SD)) were enrolled in the study and started a VLCKD dietary program (Pentadiet program, Figure 1) with a total intervention duration of 24 weeks. Eleven patients were on chronic therapy known not to interfere with VLCKD treatment (Table S1). The concomitant medications of the study subjects at baseline are reported in Table S1. The indicated treatments were carried on until the end of the VLCKD program, under appropriate monitoring for possible adverse effects. The study was conducted in accordance with the guidelines of the declaration of Helsinki (http://www.wma.net/en/30publications/10policies/b3/, accessed 2 April 2021), and the study protocol was approved by the Institutional Ethics Committee (approval N°441/2011). Patients were informed about all aspects related to the study, possible benefits and risks were explained at the beginning of the study and subjects were informed about the possibility to leave the study at any time without penalty. Written informed consent was obtained from each subject before starting the VLCKD program.

### 2.2. Clinical Procedures

The overall duration of the study was 24 weeks, divided into 4 sequential phases: two “active phases” (phases 1 and 2) and two “stabilization phases” (phases 3 and 4) (Figure 1). Each phase had a standard duration, and the daily plan included 3 main meals (breakfast, lunch, dinner) and 1 snack in the afternoon in all phases. All low-carbohydrate foods (Protiligne) and a food supplement (PentaCal) used in the VLCKD program were provided by New Penta srl (Milan, Italy). The average daily food intake, including pre-prepared meals (Protiligne, Table S2B), varied according to each phase. As reported in Table S2, the energy and macronutrient content of meal replacement portions were within the indicated range, and varied according to each specific type (i.e., soups, cakes, meat plates, etc.). Thus, during each phase of the program, the daily target of energy and macronutrients was reached combining different meal replacement portions and the allowed foods. The daily intake of protein, carbohydrate, linoleic acid, γ-linoleic acid and micronutrients during all the phases of the VLCKD program was above the minimum content recommended by EFSA, according to a specific Scientific Opinion (https://www.efsa.europa.eu/it/efsajournal/pub/2271, accessed 2 April 2021). Patients were instructed to drink not less than 1.5–2 L of water daily and to avoid ingestion of any sweets, sugarfree chewing gums and soft drinks, herbal tea with fruit, and preserved vegetables. The program included the use of a vitamin and mineral supplement (PentaCal, Table S2A) during phases 1 and 2. At the end of the study, a compliance survey was submitted to all patients.

The efficacy of VLCKD was assessed by evaluating anthropometric measures (height, weight, BMI, WC and HC), SBP/DBP, HR and glucose metabolism markers, whereas liver and kidney function biomarkers were assessed as safety parameters.

### 2.3. Blood and Urinary Biochemistry

Before starting the VLCKD program and at the end of phases 2 and 4, urine and fasting blood samples from an antecubital vein were collected at 8:00–10:00 a.m. after an overnight fast. The following haematological and biochemical parameters, used as efficacy and safety end-points, were evaluated using standard automated clinical procedures (Cobas system, Roche, Italy): complete blood count, electrolytes (chloride, potassium, calcium, magnesium, sodium), fasting plasma glucose (FPG) and insulin, HbA1c, plasma protein concentration, lipids (total cholesterol (TC), HDL cholesterol (HDL-C) and triglycerides (TG)), uric acid, blood urea nitrogen, creatinine, alanine transferase (AST), aspartate transaminase (AST), γ-glutamyl transpeptidase (γ-GT), high-sensitivity C-reactive protein (hs-CRP) and TSH reflex. Urinary ketones were evaluated using Ketostix strips (Bayer, Germany). All biochemical analyses were conducted in 3 certified clinical laboratories in the Milan area. All samples from each participating subject were collected and analyzed in the same laboratory. LDL cholesterol (LDL-C) was calculated according to the Friedewald formula [26]. The homeostasis model assessment of insulin resistance (HOMA-IR) index was calculated as follows: HOMA-IR = (fasting glucose (mmol/L) × insulin (mU(mL))/22.5) [27]. The triglyceride-glucose (TyG) index was calculated as follows: ln (TG × FPG/2). Creatinine clearance was calculated according to the Cockroft-Gault formula [28].

### 2.4. Statistical Analysis

Sample size calculation. A sample size of at least 26 subjects in the study group achieves 90% power to detect a reduction of 10% in body weight vs. population of obese women in the same range of BMI (from 27 to 37 kg/m^2^; mean body weight = 85 kg; standard deviation = 13 kg), with a type I error rate of 5%. The cardiovascular risk score was calculated according to the Framingham Risk Score using lipid values (FRS lipids) and using BMI (FRS BMI) [29] and the EAS/ESC SCORE for low-risk countries (like Italy) [30]. A per protocol analysis was performed. Quantitative variables are presented as mean values ± standard deviation, SD), while qualitative variables are presented as frequencies. Comparisons between continuous variables across visits were performed by using the non-parametric Friedman test for k mutually related samples. All reported *p*-values are based on two-sided tests and compared to a significance level of 5%. All statistical analyses were performed using IBM SPSS Statistics software package for Windows, Version 25.0. Armonk, NY, USA: IBM Corp.

## 3. Results

### 3.1. Study Population

**Figure 2:**
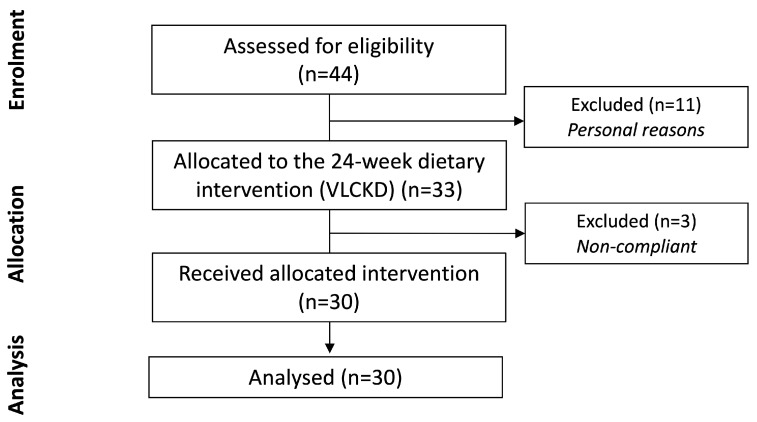
CONSORT statement flow diagram.

The study included women with upper-range overweight or grade 1–2 obesity and was conducted in a real-life setting. Among the 44 eligible patients, 11 were excluded before the start of the VLCKD program, due to personal reasons or duties, such as lack of motivation in undergoing the dietary plan or family problems (Figure 2). Therefore, 33 subjects were allocated to the VLCKD program, and, since 3 participants dropped-out during phase 1 (*n* = 2) or phase 2 (*n* = 1) by directly declaring to exit from the VLCKD program, due to lack of interest/motivation, 30 subjects completed the study (Figure 2) and their baseline data are reported in Table 1. The study subjects had a BMI of 30.9 ± 2.7 kg/m^2^, with a relevant abdominal adiposity (WC: 96.0 ± 9.4 cm), mild dyslipidemia (LDL-C: 144.0 ± 33.6 mg/dL; non HDL-cholesterol (non-HDL-C): 164.9 ± 35.7 mg/dL) and some degree of insulin resistance, as shown by a moderately elevated HOMA-IR (3.17 ± 2.67).

### 3.2. Analysis of the Ketogenetic Effect of VLCKD

The determination of urinary ketones, an indirect index of carbohydrate restriction and adherence to the proposed dietary plan based on a VLCKD approach, was performed in order to evaluate the actual presence of ketogenesis produced by dietary carbohydrate restriction during the first 2 phases of the protocol. As expected, urinary ketones were not detectable at baseline. The occurrence of dietary-induced ketogenesis, detected by the presence of urinary ketones, was observed in 78% of the patients after phase 1 and in 50% of the patients after phase 2.

### 3.3. Effect of VLCKD on Anthropometric Parameters

**Figure 3:**
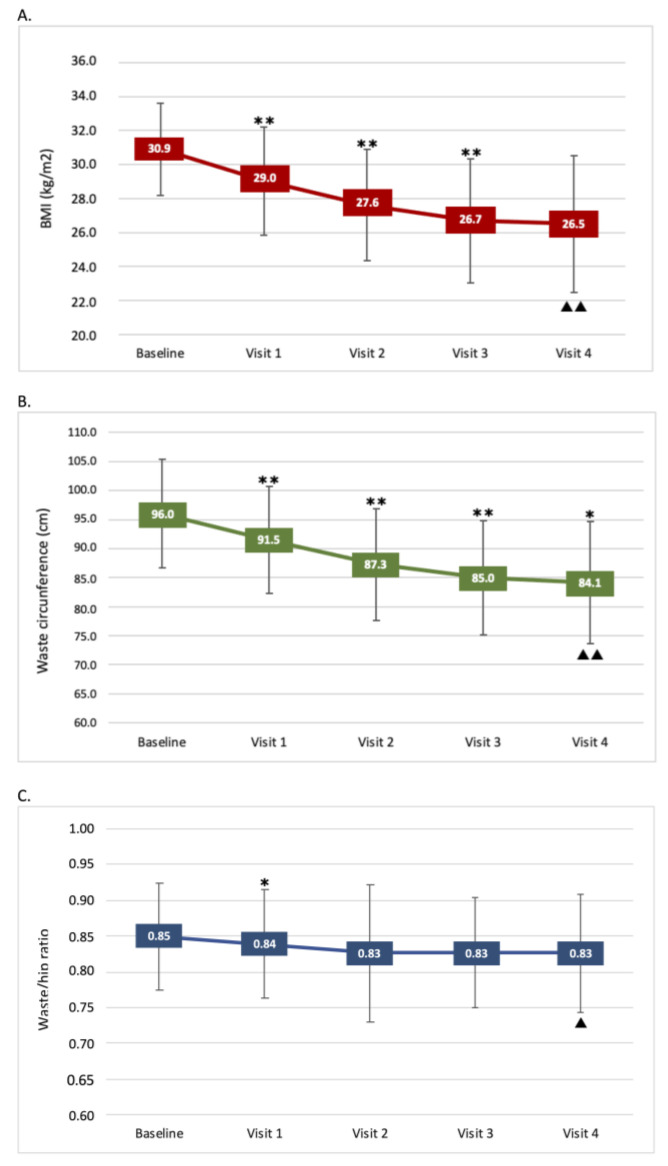
Effect of the VLCKD program on BMI, waist circumference and waist/hip ratio. (**A**) BMI changes during the 24-week program; (**B**) waist circumference during the 24-week program; (**C**) waist/hip ratio during the 24-week program. Data are mean ± SD. (*) *p* < 0.05 and (**) *p* < 0.001: *p*-value across consecutive visits. (▲) *p* < 0.05 and (▲▲) *p* < 0.001: *p*-value for trend.

**Figure 4:**
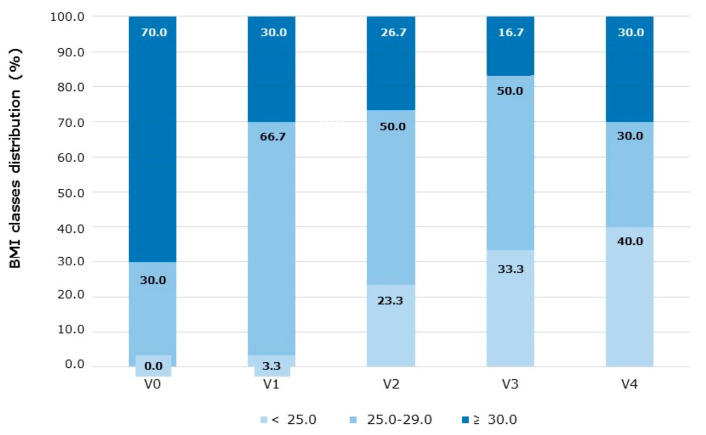
Effect of the VLCKD program on the BMI classes distribution. The relative % distribution of patients in the normal weight (<25.0 kg/m^2^), overweight (<25.0–29.9 kg/m^2^) and obese (>30 kg/m^2^) BMI classes, over the 24-week VLCKD program, is reported. V, visit.

Over the entire VLCKD program, which lasted 24 weeks, all anthropometric parameters were progressively improved, with a total significant decrease of 14.6% in body weight and BMI (Figure 3A), 12.4% in WC (Figure 3B) and 10.0% in HC, resulting in a lower (−2.7%) Waist-to-Hip ratio (WHR) (Figure 3C). It should be highlighted that the reduction of BMI and WC in a single-phase, although significant after each of them compared to the start value, was greater during phases 1–2 (BMI: −6.2% and −4.9%, WC: −4.7% and −4.6%, respectively) (Figure 3A,B), although some contribution to total weight loss was observed in all subsequent phases, leading to a cumulative 11.5 kg weight loss, on average. At the end of the VLCKD protocol, 33.3% of the participants reached a normal weight and the obesity prevalence was reduced from 70% to 16.7%. As a consequence, the overweight group rose from 30 to 50% (Figure 4).

### 3.4. Effect of VLCKD on Glucometabolic and Cardiovascular Parameters

At baseline, patients enrolled in the study displayed a moderate rate of insulin resistance (HOMA-IR: 3.17 ± 2.67) (Table 1. The VLCKD program showed a specific effect on this parameter, as it was significantly reduced to 1.73 ± 1.23 (−38.0%; *p* = 0.003) at the end of phase 2, due to reduction of both plasma insulin (−35.0%; *p* < 0.001) and FPG (−8.7%; *p* = 0.002), in association with reduced HbA1c (−5.6%; *p* = 0.008), and then remained unchanged after phase 3 (Table 2). The TyG index was also significantly improved (*p* < 0.001) (Table 2). No changes in uric acid levels were observed (Table 3). SBP decreased after phase 1 (−3.5 mmHg; −2.5%; *p* = 0.006) and then remained unchanged until the end of the program. As reported above, the study subjects showed moderate baseline hypercholesterolemia (TC 223.0 ± 37.7) mg/dL). TC, TG and LDL-C were significantly reduced by the VLCKD program after phase 3, along with a significant increase of HDL-C (*p* = 0.027), resulting in reduced non-HDL-C (Table 3). The individual change of LDL-C level showed some variability since 6/30 patients displayed no changes and 6/30 had moderately increased concentrations (maximum 158 mg/dL in one case). Moreover, hsCRP (always below 0.1 mg/L; not shown) and uric acid (Table 3) concentrations did not significantly change during the intervention. At baseline, the study subjects were almost entirely at very low/low CVD risk, according to FRS lipids, FRS BMI and EAS/ESC SCORE algorithms. Interestingly, however, the BMI and lipid improvements driven by VLCKD resulted in a mean absolute reduction of these scores: FRS lipids (from 1.99 ± 1.57 to 1.53 ± 1.20), FRS BMI (from 6.23 ± 4.13 to 5.05 ± 3.12) and EAS/ESC SCORE (from 0.42 ± 0.34 to 0.36 ± 0.30), due to the specific reduction in the few with higher CVD risk.

### 3.5. Effect of VLCKD on Markers of Liver, Kidney and Thyroid Function

At baseline, the markers of liver, kidney and thyroid function were within the reference range and remained within it over the entire duration of the VLCKD program (Table 3). A significant but moderate decrease was observed for γ-GT, creatinine and creatinine clearance (Table 3).

## 4. Discussion

This study aimed at evaluating the efficacy and the safety of a multiphasic VLCKD program, conducted in a multi-center real-world setting, in women with overweight or obesity. The main objective was to assess the actual health benefits of such approach in the context of the day-by-day management of these clinical conditions. The proposed multiphasic VLCKD program turned out to be safe, according to liver, kidney and thyroid biomarkers. Moreover, in the patients who completed the program, a set of important improvements related to cardiovascular function and cardiometabolic disease risk has been accomplished.

The efficacy data obtained show that the VLCKD program resulted in a significant reduction (−14.6%) of body weight and BMI, which is also greater than the 10% threshold proposed by the obesity guidelines [15]. The average absolute reduction of BMI (−4.4 kg/m^2^) is similar to that obtained in hospital-based studies with a ketogenic phase up to 4 weeks (−4.2 kg/m^2^) or at least 4 weeks (6.2 kg/m^2^) [31]. Notably, the mean BMI value at the end of the protocol (26.5 kg/m^2^) is just above the upper end (25 kg/m^2^) of the normal range, with a reduction of subjects in the obese range from 70 to 16.7% at visit 3, but some weight regain at visit 4, leading to a final 30% obese subjects. On the other side, the percentage of subjects in the normal BMI range stably increased from 0% at baseline to 40% at the end of the VLCKD program. These findings may suggest that the health professional input is relevant not only in the initial phase of the VLCKD program but also in the last phase and the subsequent follow-up over the months and the years, in order to promote the longest time free of disease. Follow-up visits are important since, according to the obesity guidelines [15], once achieved, the body weight reduction of at least −10% or more should be maintained at least for 5 years to obtain an optimal benefit. Unfortunately, data from follow-up visits, after completion of the 6-month VLCKD program, could not be collected in this study, highlighting the relevant lack of long-term follow-up control visits in the real-world context. Possible reasons are lack of motivation, reduced synergy with the physician or the team and additional costs. In any case, this may clearly result in a long-term reduced benefit of the initial weight loss, since only one recommendation of the guidelines (weight reduction by at least −10%, but not 5 years maintenance) is fulfilled.

Interestingly, an additional important advantage of this VLCKD protocol was the marked decrease of WC, which was reduced by 11.9 cm to an average of 84.1 cm, which is even below the cut-off proposed by the harmonized criteria for metabolic syndrome [25] and in line with previous meta-analysis data [31]. Needless to say, this was a major benefit [8,32], which is reflected by the improvements of a several cardiometabolic biomarkers. In our study, we observed a reduction of SBP, TC, TG, LDL-C and a small but significant increase of HDL-C. The impact of VLCKD on LDL-C is still controversial in some instances, since it has been reported either unchanged [31] or reduced, such as, on average, in our study and in other recent studies conducted in men [33], or increased in a subset of patients (1 out of 4 patients) undergoing VLCKD [34], probably due to the impact of some gene variants [22]. These observations suggest that several factors, such as sex (our study included only women), the presence of selected gene variants, etc., may influence the individual LDL-C response to VLCKD and, indeed, also in our study we found some patients with no LDL-C changes and a few with a moderate increase of this marker. These findings then highlight the importance to evaluate LDL-C levels before and during/after a VLCKD program, making sure, when appropriate, to implement a specific diagnostic and therapeutic evaluation to assess ASCVD risk [35].

The overall reduction of CVD risk scores appears to be an important achievement of the VLCKD treatment evaluated in this study. Although the selected study cohort was already at low CVD risk at baseline, due to the female sex, no smoking, and the low-risk area (Italy) of their origin, the VLCKD program resulted in a further reduction (due to LDL-C and SBP reduction) of the SCORE CVD risk and of the FRS BMI and FRS lipids. Therefore, the VLCKD-driven improvement of several variables, either included or not in these risk algorithms, plays a role in reducing the global CVD risk.

A relevant reduction of insulin resistance, according to HOMA-IR reduction from 3.17 to 1.78 on average, represented another benefit, in line with other hospital-based studies [36]. Interestingly, subjects with HOMA-IR values above the threshold of 2, which indicates the presence of insulin resistance, were 66.7% at baseline but only 30% at the end of the protocol, suggesting that some participants did not fully improve their insulin resistance status.

These results obtained in a real-world setting thus appear comparable with those obtained in hospital-based studies and are relevant not only for body weight reduction per se but also of advantage in the overall reduction of primary CV and metabolic risk.

VLCKD may be a challenging approach for patients, especially in the first 2 phases, and requires a series of social and psychological features that may not be available to all subjects candidate to such treatment. This is reflected by a rather relevant rate of drop-out or non-compliance associated with VLCKD. Overall, 11/44 subjects either did not start our protocol and additional 3/33 (9%) dropped out within the first week of treatment, due to family reasons or lack of motivation to implement such a specific dietplan. Such drop-out rate is similar to that (7.5%) previously reported [31], suggesting that, since a VLCKD is obviously conducted as outpatients, the quality of the health personnel in our 5 clinical facilities was not substantially different from that present in research hospitals. It is important to emphasize that the maximum reduction of body weight/BMI and of WC as well as cardiometabolic improvements were achieved after completion of the entire VLCKD. Thus, it is important to avoid, especially in the real-world setting, the earlier interruption of such program after phases 1, 2 and 3, which sometimes happens due to excessively fast expectations by patients or quicker access to subsequent plastic surgery. Interestingly, some strategies to improve adherence to VLCKD in the real-world setting have been recently published [37,38]

This study has some limitations. A control group undergoing standard of care treatment (i.e., a low-calorie balanced diet) was not included, which does not allow one to compare this approach to the VLCKD one, when referring, for example, to CVD risk reduction. In this regard, a study reporting the comparison between VLCKD and standard low-calorie diet in the treatment of obesity in a hospital setting [39] showed that, over a 12-month timeframe, the VLCKD intervention was associated with much greater improvement of anthropometric parameters.

Moreover, no body composition assessment or indirect calorimetry could be conducted and no blinding was possible, nor was the compilation of a food diary was achievable. In addition, only three blood samplings were performed, along with the five visits, without the possibility to collect and store additional serum samples for additional experimental determinations (i.e., adipokines and pro-inflammatory cytokines). This precluded the opportunity for a more detailed cardiometabolic study, for example, evaluating the leptin: adiponectin ratio, which is markedly reduced by loss of adipose mass and has been shown to predict carotid intima-media thickness in males [40] or of circulating ghrelin levels [19,41]. Importantly, men and non-Caucasian subjects could not be included in this study since both are not referring in a relevant way to clinical practice for VLCKD in Italy.

The findings of this study on a multi-center VLCKD program conducted in a real-world setting in a cohort of women with overweight or obesity indicate that it is safe and effective since it results in a major improvement of cardiometabolic parameters, thus leading to benefits that span well beyond the mere body weight/abdominal adiposity reduction, as they lead to a decreased primary CVD and metabolic risk. Our data cannot however be directly extended to women with severe obesity (BMI > 37 kg/m^2^) and relevant organ complication or failure, or to the male sex, which should be the focus of specific studies. Future developments in the practical application of VLCKD, especially in real-world clinics, may include the evaluation of genomic determinants of responsiveness to VLCKD and their clinical implementation following rigorous frameworks for gene variant interpretation [34].

## Figures and Tables

**Table 1:** Baseline data (*n* = 30).

	MEAN ± SD	MINIMUM	MAXIMUM
Age (years)	49.5 ± 7.2	27	60
Weight (kg)	81.8 ± 10.9	63.0	104.6
Height (m)	1.62 ± 0.07	1.48	1.78
BMI (kg/m^2^)	30.9 ± 2.7	26.96	36.06
Waist circumference (cm)	96.0 ± 9.4	80.0	114.0
Hip circumference (cm)	113.1 ± 7.7	100.0	130.0
Waist-to-hip ratio	0.85 ± 0.08	0.72	1.04
SBP (mmHg)	127.2 ± 10.2	110	160
DBP (mmHg)	81.5 ± 8.9	60	100
Heart rate (bpm)	69.4 ± 6.3	52	80
FPG (mg/dL)	95.1 ± 15.6	73	155
HbA1c (mmol/mol)	36.98 ± 5.19	30.05	58.40
Insulin (mU/L)	12.65 ± 7.31	3.00	39.60
HOMA-IR	3.17 ± 2.67	0.64	15.16
Total cholesterol (mg/dL)	223.0 ± 37.7	159	339
HDL-cholesterol (mg/dL)	58.0 ± 12.9	37.3	82.7
Non HDL-cholesterol (mg/mL)	164.9 ± 35.7	101.3	289.3
Triglycerides (mg/dL)	104.7 ± 41.4	44	208
LDL-cholesterol (mg/dL) (*)	144.0 ± 33.6	80.1	248.3
Uric acid (mg/dL)	4.6 ± 1.0	3.1	6.6
AST (mg/dL)	18.5 ± 4.6	12	32
ALT (mg/dL)	20.5 ± 12.2	8	63
γ-GT (mg/dL)	21.0 ± 8.6	10	46
Creatinine (mg/dL)	0.74 ± 0.13	0.44	0.98
Creatinine clearance (mL/min)	122.40 ± 33.09	73.65	221.49
BUN (mg/dL)	33.39 ± 8.62	22.40	51.00
TSH (mUI/L)	2.38 ± 0.80	1.01	3.70

BMI, body mass index; SBP, systolic blood pressure; DBP, diastolic blood pressure; FPG, fasting plasma glucose; HbA1c, glycosylated hemoglobin; HOMA IR, homeostatic model assessment for insulin resistance; AST, aspartate transaminase; ALT, alanine transaminase; γ-GT, gamma-glutamyl transferase; BUN: blood urea nitrogen; TSH, thyroid-stimulating hormone. (*) calculated by the Friedewald formula.

**Table 2:** Effect of VLCKD on glucometabolic parameters.

		Mean ± SD	Absolute Change (% Change)	*p*-Value *
FPG (mg/dL)	Baseline	95.1 ± 15.6	−9.3 (−9.8)	0.001
	Visit 2	85.9 ± 12.1		
	Visit 3	85.8 ± 11.9		
HbA1c (mmol/mol)	Baseline	36.98 ± 5.19	−2.47 (−6.0)	0.001
	Visit 2	34.75 ± 2.82		
	Visit 3	34.51 ± 3.14		
Insulin (µU/mL)	Baseline	12.65 ± 7.31	−4.72 (−37.3)	0.001
	Visit 2	7.73 ± 4.92		
	Visit 3	7.93 ± 6.10		
HOMA-IR	Baseline	3.17 ± 2.67	−1.39 (−43.8)	0.001
	Visit 2	1.73 ± 1.23		
	Visit 3	1.78 ± 1.82		
TyG index	Baseline	8.43 ± 0.45	−0.41 (−4.9)	0.001
	Visit 2	8.05 ± 0.38		
	Visit 3	8.02 ± 0.49		

FPG, fasting plasma glucose; HbA1c: glycosylated hemoglobin; HOMA-IR: Homeostatic Model Assessment for Insulin Resistance; TyG index, triglyceride-glucose index; * Friedman test for k mutually related samples.

**Table 3:** Effect of VLCKD on cardiovascular, lipid and safety parameters.

		Mean ± SD	Absolute Change (% Change)	*p*-Value *
SBP (mmHg)	Baseline	127.2 ± 10.2	−3.5 (−2.8)	0.006
	Visit 1	123.3 ± 9.8		
	Visit 2	123.2 ± 9.6		
	Visit 3	121.4 ± 8.4		
	Visit 4	123.7 ± 9.6		
DBP (mmHg)	Baseline	81.5 ± 8.9	−3.5 (−4.3)	0.211
	Visit 1	80.1 ± 8.2		
	Visit 2	80.0 ± 9.3		
	Visit 3	78.6 ± 9.0		
	Visit 4	78.0 ± 8.6		
HR (bpm)	Baseline	69.4 ± 6.3	−0.3 (−0.4)	0.021
	Visit 1	72.0 ± 6.7		
	Visit 2	69.7 ± 5.0		
	Visit 3	70.4 ± 6.8		
	Visit 4	69.1 ± 12.9		
TC (mg/dL)	Baseline	223.0 ± 37.7	−13.2 (−5.9)	0.000
	Visit 2	194.8 ± 30.7		
	Visit 3	209.7 ± 28.4		
HDL-C (mg/dL)	Baseline	58.0 ± 12.9	3.3 (5.7)	0.000
	Visit 2	52.7 ± 12.7		
	Visit 3	61.7 ± 13.0		
TG (mg/dL)	Baseline	104.7 ± 41.4	−27.1 (−25.9)	0.000
	Visit 2	78.4 ± 29.1		
	Visit 3	77.6 ± 31.1		
LDL-C (mg/dL) (°)	Baseline	144.0 ± 33.6	−11.2 (−7.8)	0.000
	Visit 2	126.4 ± 23.4		
	Visit 3	132.8 ± 23.7		
non HDL-C (mg/dL)	Baseline	164.9 ± 35.7	−16.5 (−10.1)	0.000
	Visit 2	142.1 ± 23.4		
	Visit 3	148.4 ± 24.4		
TG/HDL-C	Baseline	1.97 ± 1.14	−0.6 (−30.5)	0.001
	Visit 2	1.62 ± 1.05		
	Visit 3	1.35 ± 0.73		
Uric acid (mg/dL)	Baseline	4.6 ± 1.0	−0.3 (−6.5)	0.093
	Visit 2	4.5 ± 1.1		
	Visit 3	4.3 ± 1.1		
AST (UI/L)	Baseline	18.5 ± 4.6	−0.3 (−1.6)	0.246
	Visit 2	19.5 ± 6.0		
	Visit 3	18.2 ± 5.3		
ALT (UI/L)	Baseline	20.5 ± 12.2	−1.5 (−7.3)	0.899
	Visit 2	21.4 ± 13.5		
	Visit 3	19.0 ± 9.2		
γ-GT (UI/L)	Baseline	21.0 ± 8.6	−5.1 (−24.3)	0.000
	Visit 2	16.0 ± 8.3		
	Visit 3	15.9 ± 9.1		
Creatinine (mg/dL)	Baseline	0.74 ± 0.13	−0.09 (−12.2)	0.004
	Visit 2	0.73 ± 0.13		
	Visit 3	0.65 ± 0.11		
CC (mL/min)	Baseline	122.39 ± 33.09	−4.40 (−3.6)	0.026
	Visit 2	110.32 ± 26.15		
	Visit 3	117.99 ± 25.59		
BUN (mg/dL)	Baseline	33.39 ± 8.62	2.46 (7.4)	0.092
	Visit 2	35.35 ± 6.22		
	Visit 3	35.85 ± 8.94		
TSH (mUI/L)	Baseline	2.40 ± 0.77	−0.09 (−3.8)	0.629
	Visit 2	2.21 ± 0.88		
	Visit 3	2.31 ± 0.86		

SBP, systolic blood pressure; DBP, diastolic blood pressure; HR, heart rate; TC, total cholesterol; HDL-C, HDL-cholesterol; TG, triglycerides; LDL-C, LDL-cholesterol; non-HDL-C, non-HDL-cholesterol; AST, aspartate transaminase; ALT, alanine transaminase; γ-GT, gamma-glutamyl transferase; CC, creatinine clearance; BUN, blood urea nitrogen; TSH, thyroid-stimulating hormone; (°) calculated by the Friedewald formula; * Friedman test for k mutually related samples.

## Data Availability

The data presented in this study are available on request from the corresponding author. The data are not publicly available due to privacy reasons.

## Supplementary Materials

The following are available online at https://www.mdpi.com/article/10.3390/nu13061804/s1, Table S1: Concomitant medications; Table S2A: Composition of the PentaCal supplement; Table S2B: Composition of the Protiligne meal replacement (range of content per portion).
