# Supplementary material for: Reduction of Cardio-Metabolic Risk and Body Weight through a Multiphasic Very-Low Calorie Ketogenic Diet Program in Women with Overweight/Obesity: A Study in a Real-World Setting
Source: Nutrients. 2021 May 26;13(6):1804. doi: 10.3390/nu13061804 (PMC8230107; doi:10.3390/nu13061804)
Supplement: Supplementary file 1 [file nutrients-13-01804-s001.zip › nutrients-1196613-SI.pdf]

Supplementary tables

Table S1: Concomitant medications.

Medication	Subjects (%)
ACE-I/ARB	46.7
beta-blockers	20
diuretics	20
calcium antagonists	6.7
allopurinol	23.3
proton-pump inhibitors	10
other drugs	46.3

ACE-I, angiotensin-converting-enzyme inhibitor; ARB, Angiotensin Receptor Blockers.

Table S2: (**A**) Composition of the PentaCal supplement.

	Daily Dose
Total citrates	3000 mg
Potassium	534 mg
Magnesium	375 mg
Selenium	83 μg
Vitamin C	120 mg
Vitamin E	18 mg
Vitamin B6	3 mg
Vitamin B2	2.4 mg
Vitamin B1	2.1 mg
Vitamin A	1200 μg
Folic Acid	400 μg
Vitamin B12	3.75 μg

Table S2. (**B**) Composition of the Protiligne meal replacement (range of content per portion).

Carbohydrates (g)	1.4–4.2
Including simple sugars (g)	0.2–0.5
Protein (g)	17.3–28.2
Total Fat (g)	0.16–4.6
Energy (kCal)	101–160
